# Effect of Plasma Treatment on Multi-Walled Carbon Nanotubes for the Detection of H_2_S and SO_2_

**DOI:** 10.3390/s120709375

**Published:** 2012-07-09

**Authors:** Xiaoxing Zhang, Bing Yang, Xiaojing Wang, Chenchen Luo

**Affiliations:** State Key Laboratory of Transmission & Distribution Equipment and Power System Safety and New Technology, Chongqing University, Chongqing 400030, China

**Keywords:** carbon nanotubes, gas sensor, modification

## Abstract

H_2_S and SO_2_ are important characteristic gases of partial discharge (PD) generated by latent insulated defects in gas insulated switchgear (GIS). The detection of H_2_S and SO_2_ is of great significance in the diagnosis and assessment of the operating status of GIS. In this paper, we perform experiments on the gas sensitivity of unmodified multi-walled carbon nanotubes (MWNTs) and those modified by atmospheric pressure dielectric barrier discharge (DBD) air plasma at different times (30, 60 and 120 s) for H_2_S and SO_2_, respectively. The results show that the sensitivity and response time of modified MWNTs to H_2_S are both improved, whereas the opposite effects are observed for SO_2_. The modified MWNTs have almost zero sensitivity to SO_2_. Thus, the MWNTs modified by atmospheric pressure DBD air plasma present good selectivity to H_2_S, and have great potential in H_2_S detection.

## 1. Introduction

Gas insulated switchgear (GIS) has been widely used in power systems due to its compact structure, small footprint and high reliability. However, some latent insulation faults in GIS are inevitable in the progress of manufacturing, assembly and operation, resulting in different degrees of partial discharge (PD) which lead to the decomposition of sulfur hexafluoride (SF_6_) gas. SOF_2_, SO_2_F_2_, SOF_4_, SO_2_, H_2_S, and HF are produced if there are trace amounts of air and water vapor present. These gases are close to the degree and type of PD. H_2_S and SO_2_ are important characteristic gases of partial discharge produced by latent insulation defects in GIS [1,2]. Thus, the detection of H_2_S and SO_2_ has important significance in the diagnosis and assessment of the state of GIS equipment operations.

Gas sensor has been used in detecting the decomposition components of SF_6_ under PD. Since their discovery by Iijima [3], carbon nanotubes (CNTs) have received considerable attention as active elements for gas-sensing devices due to their rich hole structure and high surface to volume ratio; they are also characterized by conductance that can be easily perturbed by interaction with gas molecules [4–10]. Compared with the conventional gas sensors, CNT-based gas sensors possess outstanding properties, such as higher sensitivity, faster response, lower operating temperature, smaller size and detectability of larger variety of gas species [11].

Improving sensitivity and selectivity is important for CNT-based gas sensors [12–14]. In 2003, Qi *et al*. [15] reported that CNTs coated with Nafion and poly-ethyleneimine show selectivity. In a NO_2_ and NH_3_ filled environment, CNTs coated with poly-ethyleneimine can detect NO_2_, with a concentration below 1 ppb, excluding the interference of NH_3_. Moreover, when CNTs are coated with Nafion, they can detect NH_3_, excluding the interference of NO_2_. In 2010, Molnar *et al*. [16] used CNTs to detect environmentally unfriendly gases, including N_2_O, NH_3_, and H_2_S with the method of fluctuation-enhanced sensing, thereby achieving good selectivity. In 2011, Slobodian *et al*. [17] found that after being oxidized by acidic potassium permanganate, the multi-walled carbon nanotubes (MWNTs) detected organic vapors diethyl ether, acetone, methanol, and isopentane solution with good selectivity. The interference of H_2_S and SO_2_ using CNT-based gas sensor limits the detectability of SF_6_ decomposition components. However, there have been few reports that CNT-based gas sensor showed good selectivity to H_2_S and SO_2_, respectively.

Low-temperature plasma surface treatment can modify the surface of materials effectively. It can change the surface morphology and chemical composition of MWNTs [18–20]. Atmospheric pressure dielectric barrier discharge (DBD) is a method for producing low-temperature plasma. Low-temperature plasma produced by DBD has good modification effect on materials, and has been widely used in the area of material modification [21,22]. This approach can generate large volume and high energy density low temperature plasma at atmospheric pressure ranging from 10^4^ Pa to 10^6^ Pa and broad frequency ranging from 50 Hz to 10^6^ Hz. Furthermore, it is simple and does not require expensive vacuum equipment; it also does not generate pollution and can even save energy.

In this paper, the surface of the MWNTs is modified by atmospheric pressure air DBD plasma, after which gas sensors based on MWNTs are fabricated. The experimental results show that after modification by DBD, the sensitivity and response time of MWNTs gas sensor to H_2_S, the concentration of which is 50 ppm, are improved greatly. Moreover, the MWNTs gas sensor exhibits no sensitivity to SO_2_, indicating that the modified MWNTs show good selectivity to H_2_S.

## 2. Experimental Section

### 2.1. Materials

MWNTs used in this paper were purchased from the Chengdu Institute of Organic, Chinese Academy of Sciences and were grown by chemical vapor deposition (CVD) method. The tube diameter is 20∼30 nm, length 10∼30 μm, purity >95%, and catalyst residue (ash) <1.5 wt%. Due to the cluster effect, they required pretreatment before modification so that the cluster MWNTs can be spread out evenly and achieve better modification effect. First, the MWNTs were placed into a beaker containing the appropriate ethanol solution, after which the beaker was placed in an ultrasonic bath for an hour. Finally, MWNTs was filtered out of the solution using filtration paper with a pore size of 0.1 μm. This step was performed to separate the MWNTs from the solution. Through this process, MWNTs can be spread better after pretreatment.

### 2.2. Surface Modification Experiment

**Figure 1.**
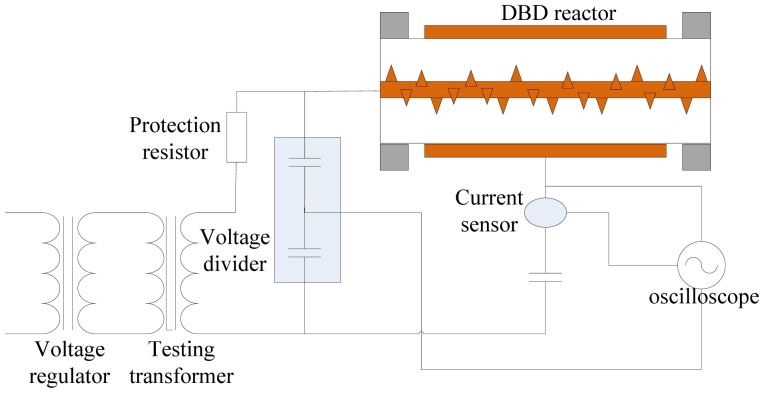
The scheme of the modifying experiment setup.

In this paper, MWNTs were treated by atmospheric pressure DBD plasma for surface modification. Air was used as precursor gas for plasma in the laboratory, and the experimental temperature was 25 °C. The scheme of the surface modification experiment setup is shown in Figure 1. The frequency of power excitation ranges from 16 kHz to 30 kHz, and the voltage amplitude was adjusted continuously in a range from 0 kV to 20 kV. Supply voltage waveform was collected by the high voltage probe P6015A (attenuation ratio 1,000). Transimission charge in discharge space was obtained by 2,000 pF capacitor in series in the circuit indirectly. The oscilloscope was a Tektronix model DPO4054.

The main body of the reactor in the modifying experiment setup was a cylindrical quartz tube. A 200 mm long copper strip was used as grounding electrode and wrapped around the outer wall of the quartz glass tube. A fixed coaxial high voltage copper rod was placed in the quartz glass tube and needle-shaped copper prick electrodes were placed in an array on the copper rod.

Resonance occurs when the power frequency is 21 kHz in Figure 1. At this frequency, cycle transmission charge, discharge power and efficiency of energy injection are all at maximum. Therefore, the frequency and peak to peak voltage of the power supply were chose to be 21 kHz and 16.4 kV, respectively.

**Figure 2.**
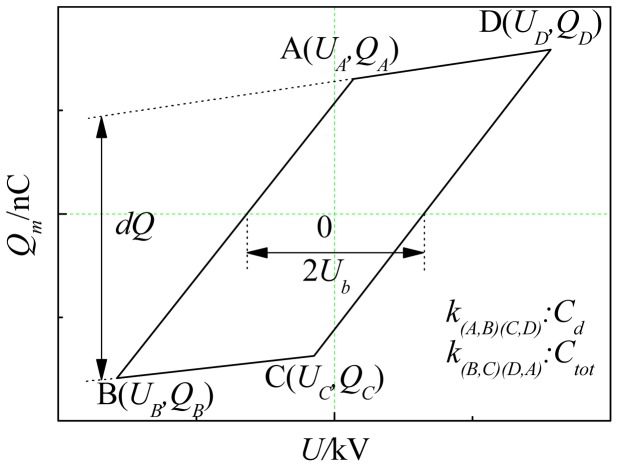
Expected Lissajous curve.

The load characteristic of DBD reactor was capacitive, and the discharge process can be modeled as capacitor charge-discharge process. The voltage U_m_ across the capacitor C_m_ was proportional to tansimission charge in discharge space Q_m_. The supply voltage and U_m_ measured by high voltage probe were added to the y-x axis of the oscilloscope, so the Lissajous curve [23] can be obtained (in Figure 2). d_Q_ in the figure was transimission charge in half cycle, and U_b_ was starting discharge voltage. DBD cycle transimission charge and discharge power can be calculated approximately by the following equations:

(1) $$Q_{m}=2(Q_{A}-Q_{B})$$

(2) $$P=\frac{1}{T}\int_{0}^{T}u\cdot i_{m}dt=f\int_{0}^{T}u\cdot C_{m}\cdot \frac{du_{m}}{dt}\cdot dt=f\oint u\cdot dq_{m}=fS$$

**Figure 3.**
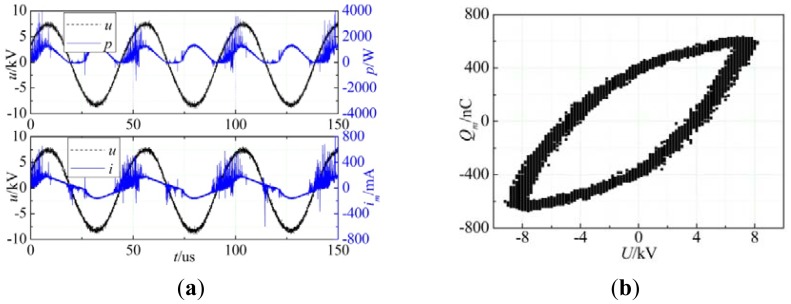
Discharge waveforms and Lissajous curve (**a**) voltage and current waveform and voltage instantaneous power waveform; (**b**) Lissajous curve.

The measured voltage and current waveform, voltage instantaneous power waveform and Lissajous curve in modified experimental conditions were shown in Figure 3. According to the equations (1) to (2), the cycle transmission charge was 1,159 nC and discharge power was 114.3 W.

After treatment, the MWNTs were distributed as thinly as possible on the bottom of the cylindrical quartz glass tube. All MWNTs were evenly distributed in the region wrapped up by the copper strip. This is because the discharge in this region is more intense, thus improving the plasma modification effect. The MWNTs were treated for 30, 60 and 120 s. In the end, we obtained MWNTs processed for different duration.

### 2.3. Sensitivity Measurement

**Figure 4.**
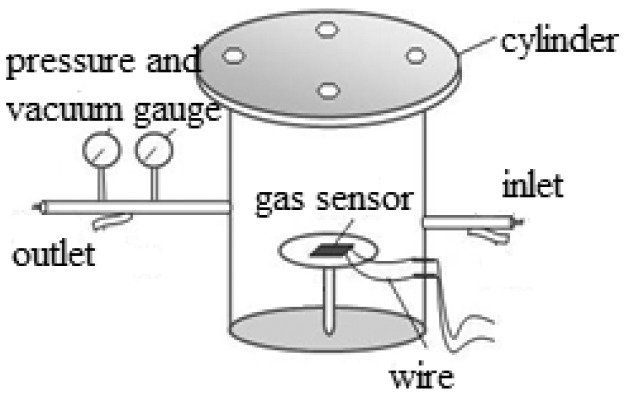
The scheme of the detecting chamber.

The substrate where the MWNTs were deposited on, was a interdigital electrodes printed circuit board with area 5 mm × 10 mm, thickness of electrodes 30 μm, space between the electrodes 1 mm. The MWNTs modified by DBD plasma were put into a beaker containing the appropriate ethanol solution, after which they were made to undergo ultrasonic treatment for 1 h. Drops of the mixed solution were dropped on the surface of the substrate. Finally, the substrates coated with MWNTs were placed in oven and baked at 80 °C for 2 h. The process was repeated for several times until uniform MWNTs film was prepared on the surface. According to this method, we fabricated four kinds of MWNTs-based gas sensors, including plasma-modified MWNTs (*i.e.*, those prepared at three different times of 30, 60, and 120 s) and untreated MWNTs. The homemade system mainly includes gas chamber, impedance and test gas. The scheme of the detection chamber is shown in Figure 4. The gas amount is controlled by gas flow meter, and the gas flow rate is about 20 sccm.

The resistance of the MWNTs gas sensor was measured in the gas experiment. The concentrations of H_2_S and SO_2_ used in the experiment were both 50 ppm. The experiment was performed at approximately 25 °C. The sensor sensitivity S is defined as:

$$S=\frac{\left|R-R_{0}\right|}{R_{0}}\times 100\%$$

where *R* and *R_0_* are the values of resistance measured in the presence of gas and vacuum, respectively.

## 3. Results

**Figure 5.**
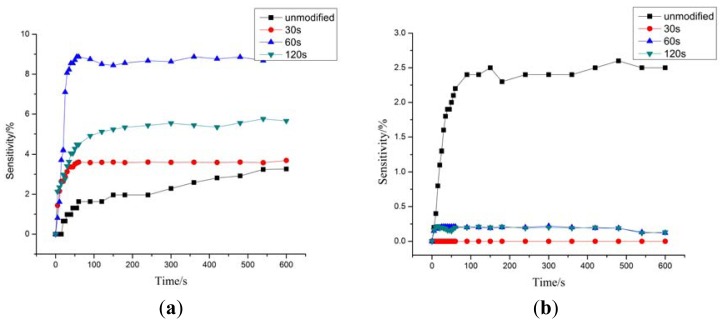
Response of MWNTs-based gas sensors to (**a**) H_2_S; (**b**) SO_2_.

We used four different kinds of MWNTs-based gas sensors (untreated MWNTs and MWNTs modified by plasma for 30, 60, and 120 s) to detect H_2_S and SO_2_ whose concentrations were both 50 ppm. The gas response curves are shown in Figure 5(a,b).

It can be seen from Figure 5(a) that the sensitivities of the untreated MWNTs and those modified by plasma for 30, 60, and 120 s to H_2_S are 3.2%, 3.6%, 8.8% and 5.6%, respectively. After plasma modification, the sensitivities of MWNTs are all enhanced, with the MWNTs treated for 60 s exhibiting the best sensitivity among them. In fact, they are 2.75 times more sensitive compared with the untreated MWNTs. From Figure 5(a), the response time of MWNTs-based gas sensors to H_2_S has also been improved greatly after treatment.

The sensitivity of untreated MWNTs to SO_2_ is about 2.5%, whereas the modified MWNTs have become less sensitive to SO_2_. The sensitivity of all modified MWNTs (those treated for 30, 60, and 120 s) to SO_2_ is almost zero. Comparing Figure 5(a,b), the MWNTs modified by plasma show different sensitive changes to H_2_S and SO_2_. The modified MWNTs not only enhanced sensitivity to H_2_S, they also reduced the response time greatly. However, it is no longer sensitive to SO_2_.

**Figure 6.**
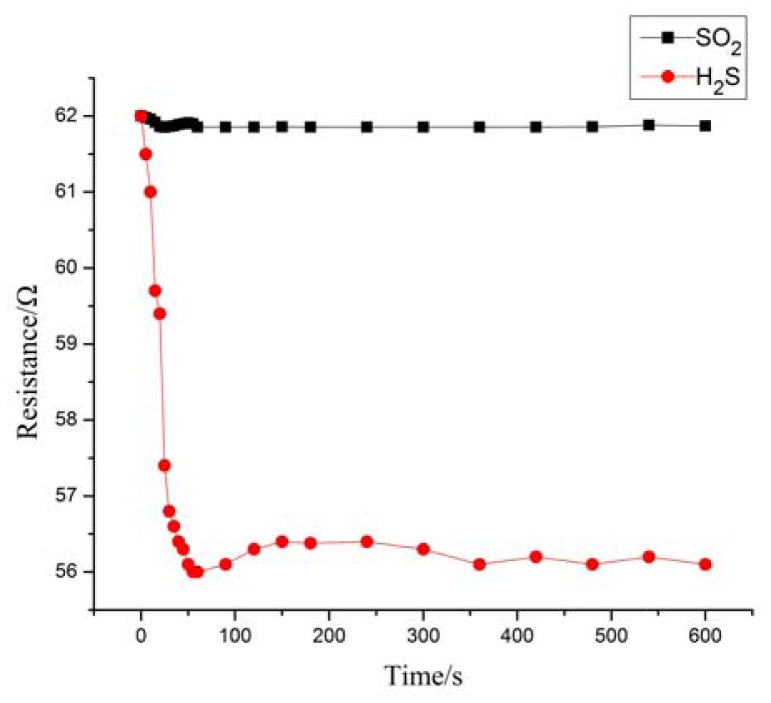
Resistance change of MWNTs modified by plasma at 60 s.

Figure 6 shows the resistance changing tendency of MWNTs modified by plasma at 60 s; the resistance of the plasma-modified MWNTs decreased in our measurement. This result shows that the air plasma-modified MWNTs exhibit n-type behavior. The majority carrier of n-type MWNTs is electron. When reducing gas interacts with n-type MWNTs, there are electrons transferring from reducing gas to MWNTs, and the number of electrons of MWNTs will increase [11,24,25]. That means the conductance will increase. In other words, the resistance will decrease.

**Figure 7.**
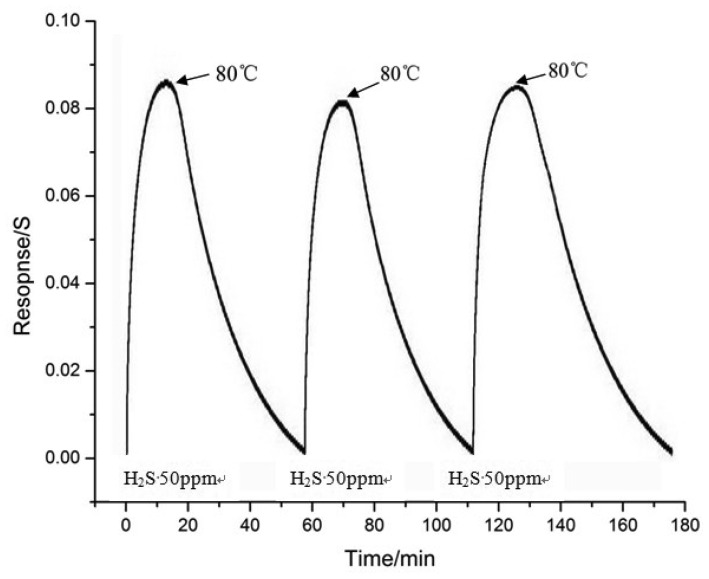
MWNTs-based gas sensors reversibility testing curve.

To study the recovery of the gas sensor, the sensor which treated by plasma for 60 s was heated immediately after turning off the H_2_S gas. The sensor was exposed to 50 ppm H_2_S at room temperature and for the recovery process the sensor was heated to 80 °C. This procedure was repeated for several times as shown in Figure 7. In Figure 7, the gas sensor was heated to about 80 °C for about 40 min, and the resistance of the MWNTs gas sensor almost recovered to the initial resistance. In conclusion, the gas sensing capacity of the sensor is recoverable.

## 4. Analysis and Discussion

We used some characterization tools to analyze the plasma-modified MWNTs.

### 4.1. FESEM Images

**Figure 8.**
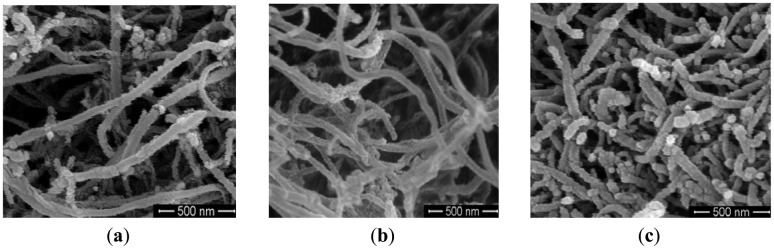
SEM images of the unmodified and modified MWNTs (**a**) unmodified; (**b**) and (**c**) modified for 60 s.

The morphologies of the plasma-modified MWNTs were characterized by field emission scanning electron microscope (FESEM). Figure 8(a) is SEM image of the unmodified MWNTs. Figure 8(b,c) are SEM images of modified MWNTs, and the two figures come from the different areas of modified MWNTs.

It can be seen from Figure 8(a) that there are many amorphous carbons and residual catalysts on the surface of untreated MWNTs. Figure 8(b) shows that the amorphous carbon and residual catalysts on the surface of MWNTs are apparently removed after plasma modification. Figure 8(c) shows that part of the MWNTs has been slightly damaged after plasma treatment. There are a small amount of surface defects, but the structural integrity has not been destroyed. Both the removal of amorphous carbons and residual catalysts on the surface of MWNTs and a small amount of surface defects may contribute to gas adsorption. Thus, the sensitivity of the modified MWNTs is enhanced greatly.

### 4.2. Analysis of Fourier Transform Infrared Spectroscopy

**Figure 9.**
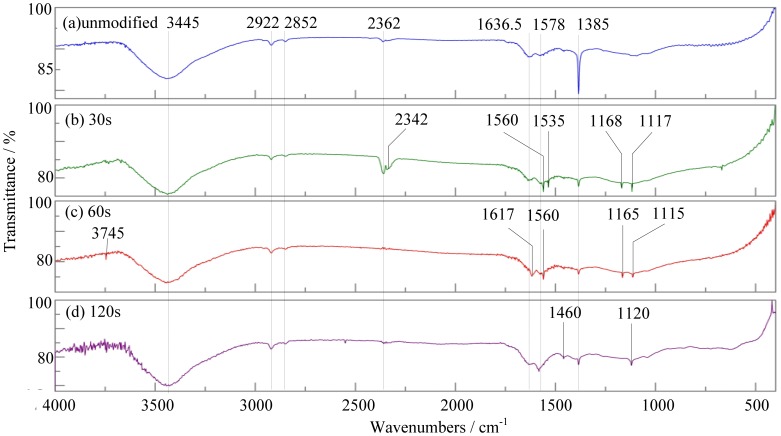
Infrared spectrums of unmodified and modified MWNTs.

Important chemical information regarding the incorporated functional groups was characterized by Fourier transform infrared spectroscopy. Figure 9 shows the infrared spectra of the untreated MWNTs and MWNTs plasma-treated MWNTs.

Comparing Figure 9(a) to 9(d), we can see that the adsorption bands they have in common are 3,445 cm^−1^, 2,922 cm^−1^, and 2,852 cm^−1^, where 3,445 cm^−1^ corresponds with the stretching vibration of hydroxyl, and 2,922 cm^−1^, 2,852 cm^−1^ correspond to the -CH_2_ asymmetric stretching vibration and symmetric stretching vibration, respectively. The appearance of these peaks resulted from the MWNT growth process with the chemical vapor deposition method.

In Figure 9(a), the adsorption bands 1,578 cm^−1^ correspond to the stretching vibration of C=C, and the adsorption bands 1,636 cm^−1^ and 1,385 cm^−1^ correspond with the *sp^2^*-C sheet structure of MWNTs and D-band caused by defects. These adsorption bands can prove that infrared spectrum of unmodified MWNTs is a typical one for MWNTs [26].

Figure 9(b) shows the infrared spectrum of MWNTs modified by plasma for 30 s. In Figure 9(b), there are absorption bands at 1,560 cm^−1^ and 1,535 cm^−1^ which do not appear in Figure 9(a). Figure 9(c) shows the infrared spectrum of MWNTs treated by plasma for 60 s. In Figure 9(c), there are absorption bands at 1,620 cm^−1^ and 1,540 cm^−1^ which do not appear in Figure 9(a) too. The four absorption bands above are in the range of 1,620∼1,540 cm^−1^ which is the asymmetric stretching of carboxylic ion. So we can infer that carboxyl is introduced in MWNTs modified by plasma for 30 s and 60 s.

Compared with Figure 9(a), the new absorption bands appeared in Figure 9(b) to (d) also include 1,168 cm^−1^ [in Figure 9(b)], 1,165 cm^−1^ [in Figure 9(c)], 1,120 cm^−1^ [in Figure 9(d)], 1,117 cm^−1^ [in Figure 9(b)] and 1,115 cm^−1^ [in Figure 9(c)]. These absorption bands range from 1,140 to 1,110 cm^−1^ which is the characteristic stretching vibration of C-N. Thus, we can infer that the nitrogen containing groups are introduced in the treated MWNTs.

From the above analysis, some carboxyl and nitrogen-containing groups are introduced on the surface of plasma-modified MWNTs. These groups interact with the gas molecules and become the active center of gas adsorption, thus increasing the sensitivity of MWNTs.

### 4.3. Analysis of MWNT Selectivity for H_2_S and SO_2_

**Figure 10.**
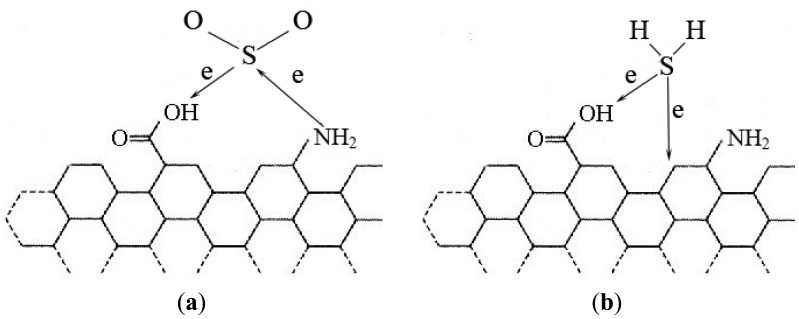
Schematic of modified MWNTs absorbing gas molecules (**a**) SO_2_; (**b**) H_2_S.

The modified MWNTs showed higher sensitivity to H_2_S and greatly reduced response time, while they showed no sensitivity to SO_2_. This is probably due to the following reason: it is known that SO_2_ shows both oxidizing and reducing behavior. According to the analysis above some carboxyl and nitrogen-containing groups are introduced onto the surface of plasma-modified MWNTs. When SO_2_ interacts with modified MWNTs [seen in Figure 10(a)], there are electrons transferring from SO_2_ to MWNTs because of the weak carboxyl oxidizability. There are also electrons transferring from MWNTs to SO_2_, because the N atoms are electron rich. When the two processes above achieve dynamic equilibrium, there are no electrons transferring between SO_2_ and MWNTs in general. So the MWNTs modified by plasma show no sensitivity to SO_2_.

The addition of nitrogen-containing groups has almost no effect on the MWNT adsorbtion of H_2_S [27]. When H_2_S interacts with the modified MWNTs [seen in Figure 10(b)], it doesn't interact with the nitrogen-containing group. However, the carboxyl on the MWNTs surface may improve MWNTs gas sensitivity to H_2_S. The reason is that these carboxyl groups can provide more adsorption sites for H_2_S molecules. In addition, carboxyl shows weak oxidizability, so the amount of the charges transferring from H_2_S to MWNTs will increase greatly, leading to the resistance of the MWNTs decreasing much more. Therefore, the modified MWNTs show higher sensitivity to H_2_S.

## 5. Conclusions

In this paper, MWNTs grown by the CVD method are modified by atmospheric pressure DBD air plasma and are used as gas-sensitive materials. We performed experiments on the gas sensitivity of the unmodified and modified MWNTs to 50 ppm H_2_S and 50 ppm SO_2_ respectively. The results show that the sensitivity of modified MWNTs to H_2_S is enhanced 2.75 times, and the response time to H_2_S greatly reduced. However, the sensitivity of modified MWNTs to SO_2_ exhibits the opposite effect. The MWNTs are almost no longer sensitive to SO_2_. Thus, the MWNTs modified by atmospheric pressure DBD air plasma presented good selectivity to H_2_S, and have great potential value in the detection of this gas.
